# Agreement of self-reported items and clinically assessed nerve root involvement (or sciatica) in a primary care setting

**DOI:** 10.1007/s00586-012-2398-5

**Published:** 2012-07-03

**Authors:** Kika Konstantinou, Martyn Lewis, Kate M. Dunn

**Affiliations:** Arthritis Research UK Primary Care Centre, Primary Care Sciences, Keele University, Staffordshire, ST5 5BG UK

**Keywords:** Sciatica, Diagnostic accuracy, Self-report, Epidemiology, Low back pain

## Abstract

**Introduction:**

We analysed baseline measures from an RCT involving adults with low back pain (LBP) with or without referred leg pain, to identify self-report items that best identified clinically determined nerve root involvement (sciatica).

**Methods:**

Potential indicators of nerve root involvement were gathered using a self-reported questionnaire. Participants underwent a standardised physical examination on the same day as questionnaire completion. Self-reported items were compared to a reference standard (clinical diagnosis) using sensitivity, specificity, predictive values, likelihood ratios (LRs), the area under the receiver operating characteristic curve and logistic regression. Two reference standards are presented: one based on a clinical diagnosis of nerve root problems and excluding possible/inconclusive cases (referred to as a *confirmatory* reference), and the other being inclusive of possible/inconclusive cases (referred to as an *indicative* reference).

**Results:**

Pain below knee was the best single item for diagnostic accuracy with an area under curve (AUC) of 0.67–0.68, which however is slightly less than the ‘acceptable discrimination’. A cluster of three items, including distribution of pain below the knee, leg pain that is worse than back pain, and feeling of numbness or pins and needles in the leg, did improve discrimination to an ‘acceptable’ level with an AUC of 0.72–0.74 in relation to confirmatory and indicative references, respectively. However, the likelihood ratios from the models were reflective of a ‘small’ amount of discrimination.

**Conclusion:**

In this primary care population seeking treatment for LBP with or without leg pain, we found no clear set of self-report items that accurately identified patients with nerve root pain. When accurate case definition is important, clinical assessment should be the method of choice for identifying LBP with possible nerve root involvement.

## Introduction

Low back-related leg pain or sciatica is one of the common variations of low back pain (LBP) [1, 2].

The literature suggests that the presence of sciatica is responsible for a poor prognosis in LBP patients [3–6]. Although definitions of sciatica used in epidemiological surveys and clinical practice vary, sciatic pain is generally defined as pain radiating to the leg, normally below the knee and into the foot and toes with varying neurological findings [7].

A recent review of sciatica prevalence studies reported a substantial variation in estimates ranging from 1.6 to 43 % [8]. The definition of sciatic symptoms seemed to explain most of the variation. This is the case also for defining back pain prevalence, leading to a recent consensus study towards standardisation of back pain definitions for use in prevalence studies so that heterogeneity in findings is minimised [9]. Dionne et al. [9] reported that in back pain research sciatica prevalence was important and suggested that in self-report studies ‘sciatica’ should be replaced by ‘pain that goes down the leg’. It is also suggested that ‘pain below the knee’ is a good proxy for clinically diagnosed sciatica and a number of studies using self-reported information have used ‘pain below the knee’ for defining sciatica [10–14], although other studies have employed more stringent self-report definitions such as ‘pain radiating to the leg(s) that worsens with coughing or sneezing’ [15]. At present, it is not known whether self-reported symptoms of sciatica correlate with the clinical diagnosis or whether ‘pain below the knee’ or ‘pain that goes down the leg’ is a reasonable proxy for the presence of sciatic symptoms.

As sciatica is considered a poor prognostic indicator in back pain presentations and may also require a different therapeutic approach to simple back pain [16, 17], accurate definitions are important for estimates of prevalence and natural history and for evaluating treatment outcome according to presentation in epidemiological studies.

The purpose of this study was twofold: to assess the agreement between self-reported leg pain and clinically defined sciatica (nerve root involvement) and if necessary to identify and assess the accuracy of an optimum cluster of self-report items for the diagnosis of sciatica.

## Methods

### Subjects and design

Patients with LBP with or without leg pain participated in a randomised controlled trial (RCT) investigating effectiveness of physiotherapy back pain treatments in primary care. All the participants were referred by their GP. Out of 851 RCT participants, 511 reported low back with pain radiating to the leg(s) and had complete data on self-reported items and recorded diagnosis of their leg pain. These 511 participants formed the sample of this analysis. Details of the RCT protocol have been reported elsewhere [18].

### Patient self-reported items

The participants completed a self-administered questionnaire at baseline on self-report measures of leg pain of spinal origin capturing area of pain, frequency and severity, effect of coughing or sneezing, description of pain quality and the presence or absence of numbness or tingling (Table 1). The questionnaire was compiled by identifying potential self-reported indicators of nerve root involvement from the literature [7, 19, 20].

**Table 1:** Self-reported items

For this set of questions, please think about the last week
1. Has the pain from your back spread down your leg(s) in the last week? Yes–No
2. How far down your leg(s) has the pain spread in the last week? Above knee–below knee (R, L)
3. If your back pain has spread down your leg(s) in the last week, where has it spread to specifically? Buttock–thigh–calf–foot (R, L)
4. In the last week, how often have you felt the pain in your leg(s)? Constantly–nearly all the time–sometimes
5. How intense was your usual leg pain (NRS; 0—no pain, 10—pain as bad as could be)
6. If you have back pain and leg pain, which one has been the worse for you in the last week? Back pain is worse–leg pain is worse–they are both as bad
7. Thinking about the last week, does the pain in your leg(s) get worse when you cough or sneeze? Yes–No
8. How would you describe the pain you felt in your leg(s) during the last week? Like toothache–sharp–shooting–tingling–burning–other
9. Have you felt numbness or pins and needles in either of your legs or feet in the last week? Yes–No

### Clinical examination

Within 10–15 min after completing the questionnaire, all the participants underwent a clinical examination by a physiotherapist. A total of 15 physiotherapists participated. The physiotherapists were blinded to the patient self-reported items. The assessment consisted of history taking in terms of pain distribution, quality of pain, easing and aggravating factors, sensory disturbances, frequency, severity and bothersomeness of leg pain. The physical examination consisted of lumbar mobility assessment, neurological testing (myotomes, reflexes, sensation) and neural tension tests (SLR, femoral stretch). All participating physiotherapists had undergone training in the assessment of back pain with leg pain. The physiotherapists were required to classify patients’ symptoms as: (a) nerve root involvement, (b) no nerve root involvement, or (c) possible nerve root involvement but not conclusive, according to their clinical judgment based on clinical findings.

### Data analysis

The clinical diagnosis was considered as the reference standard. Two diagnostic classifications are considered, which differ in the categorisation of ‘possible nerve root involvement but not conclusive’ that is pooled with: (1) the ‘no nerve root involvement’ category—this classification (referred to as *confirmatory*) was specifically aimed to target absolute clinical cases only; (2) the ‘nerve root involvement’ category—this classification (referred to as *indicative*) aimed to target ‘possible’ as well as confirmed cases. Self-reported items were compared to the reference standard (clinical diagnosis) using sensitivity, specificity, predictive values, likelihood ratios (LRs) and the area under the receiver operating characteristic (ROC) curve (c statistic, AUC) which provides the average-weighted sensitivity/specificity across the range of scale values/categories. Point and 95 % confidence interval estimates were derived for each statistic. As well as carrying out univariable comparisons, binary logistic regression analyses were performed to evaluate the prognostic accuracy of multiple items most independently predictive of the diagnosis of clinically assessed nerve root involvement (or sciatica). The classification cut-off for a sciatica diagnosis was at the customary *P* > 0.5 for the primary analysis (though this was varied to assess the impact different cut-offs had on the discriminative ability of the multivariate model). Two types of multivariable model are presented: (1) approach 1 (full-entry model), based on the inclusion of all items observed; (2) approach 2, using manual forward selection regression (with entry of items restricted to the most significant independent variables) to identify the most efficient combinations of items for discriminating a clinical diagnosis of nerve root impingement. This latter approach consisted of different models built sequentially by adding variables one-by-one in order of predictive ability on multivariable testing.

### Performance of the diagnostic model

Greater tool discrimination is reflected by: sensitivity, specificity, predictive value and AUC closer to 1; higher positive LR; lower negative LR. Likelihood ratios (LRs) from 2 to 5 represent ‘small’ increases in the post-test probability of disease, from 5 to 10 represent ‘moderate’ increases, and above 10 represent ‘large’ increases; correspondingly 0.2–0.5 reflect ‘small’ decreases in the post-test probability, 0.1–0.2 reflect ‘moderate’ decreases and <0.1 reflect ‘large’ decreases [21, 22]. Hosmer and Lemeshow [23] provided the following classification system for the AUC: 0.7 ≤ AUC < 0.8 = ‘Acceptable discrimination’; 0.8 ≤ AUC < 0.9 = ‘Excellent discrimination’; AUC ≥ 0.9 = ‘Outstanding discrimination’. From the logistic model the Nagelkerke *R* ^2^ denotes the proportion of variance explained by the model (values closer to 1 reflect a more valid tool).

## Results

Demographic information on age and gender is presented in Table 2. On clinical examination, 37.0 % (189/511) of the patients reporting low back and leg pain were classified by the assessing physiotherapist as having nerve root pain. A further 17.0 % (87/511) were documented as ‘possible neural/inconclusive’. These numbers are the basis of the reference standard diagnostic comparisons for which the self-reported items were checked for diagnostic accuracy for sciatica.

**Table 2:** Demographics

	No (%) *n* = 511	Mean (SD)
Age, year (range 18–87)		50.5 (14.9)
Female	304 (59.5)	
Pain above knee (self-reported)	248 (48.5)	
Pain below knee (self-reported)	263 (51.5)	
Symptoms on day of physical examination:		
No pain	7 (0.7)	
Low back only	121 (23.7)	
Pain in leg	371 (72.5)	
Other	12 (2.3)	

Table 3 presents the cross-tabulated frequency data of individual self-report items versus clinical classification of nerve root involvement. Data showing the diagnostic accuracy of individual items is presented in Table 4. Sensitivity and specificity of the individual items were wide ranging—although average sensitivity/specificity (as designated by the AUC value) was above 0.6 for three items: ‘pain below knee’, ‘which pain worst’ and ‘numbness, pins and needles’ (the AUC for the other items being in the range of 0.5–0.6). Sensitivity was over 50 % for ‘pain below knee’ and ‘numbness, pins and needles’ items, and for certain cut-offs of ‘frequency of pain’, ‘severity of pain’ and ‘which pain worst’. Specificity above 50 % was observed for all items. In relation to classification 1,^1^ positive predictive values were generally in the range of 0.4–0.5 whilst negative predictive values were 0.6–0.8 (i.e. NPVs being mostly higher). In contrast, in relation to classification 2,^2^ positive predictive values were generally in the range of 0.55–0.75 whilst negative predictive values were 0.45–0.65 (i.e. PPVs being mostly higher). For most items the positive likelihood ratios exceeded 1 and negative likelihood ratios were less than 1; these being statistically significant in most cases (in relation to a null hypothesis of LR = 1). However, the likelihood ratios were small: positive likelihood ratios were mostly less than the floor marker of 2 denoting a ‘small’ LR+, and negative likelihood ratios were mostly greater than the 0.5 ceiling marker for a ‘small’ LR−.

**Table 3:** Descriptive cross-tabulated frequency data of individual self-report items versus clinical classification of nerve root involvement in patients with low back pain and leg pain

Question	Non-neural (*n* = 235)	Inconclusive (*n* = 87)	Neural (*n* = 189)
Pain below knee			
No	160 (68.1 %)	36 (41.4 %)	52 (27.5 %)
Yes	75 (31.9 %)	51 (58.6 %)	137 (72.5 %)
Frequency of pain			
Sometimes	123 (53.0 %)	36 (41.1 %)	62 (32.8 %)
Nearly all the time	60 (25.9 %)	33 (37.9 %)	81 (42.9 %)
Constant	49 (21.1 %)	18 (20.7 %)	46 (24.3 %)
Severity of pain (NRS)			
0–3	60 (26.2 %)	12 (14.0 %)	25 (13.3 %)
4–6	112 (48.9 %)	46 (53.5 %)	73 (38.8 %)
7–10	57 (24.9 %)	28 (32.6 %)	90 (47.9 %)
Which pain is worst			
Back pain	139 (59.9 %)	26 (30.2 %)	58 (30.7 %)
Both as bad	54 (23.3 %)	29 (33.7 %)	55 (29.1 %)
Leg pain	39 (16.8 %)	31 (36.0 %)	76 (40.2 %)
Coughing/sneezing			
No	187 (79.6 %)	68 (78.2 %)	124 (66.0 %)
Yes	48 (20.4 %)	19 (21.8 %)	64 (34.0 %)
Tingling			
No	189 (80.4 %)	68 (78.2 %)	123 (65.1 %)
Yes	46 (19.6 %)	19 (21.8 %)	66 (34.9 %)
Shooting pain			
No	177 (75.3 %)	54 (62.1 %)	127 (67.2 %)
Yes	58 (24.7 %)	33 (37.9 %)	62 (32.8 %)
Burning pain			
No	201 (85.5 %)	68 (78.2 %)	139 (73.5 %)
Yes	34 (14.5 %)	19 (21.8 %)	50 (26.5 %)
Sharp pain			
No	182 (77.4 %)	57 (65.5 %)	118 (62.4 %)
Yes	53 (22.6 %)	30 (34.5 %)	71 (37.6 %)
Toothache			
No	127 (54.0 %)	44 (50.6 %)	101 (53.4 %)
Yes	108 (46.0 %)	43 (49.4 %)	88 (46.6 %)
Numbness, pins and needles			
No	151 (64.3 %)	43 (49.4 %)	69 (36.5 %)
Yes	84 (35.7 %)	44 (50.6 %)	120 (63.5 %)

**Table 4:** Diagnostic accuracy of individual self-report items to identify nerve root involvement (based on clinical assessment) in patients with low back pain and leg pain

Question	Diagnostic classification	Sensitivity (95 % CI)	Specificity (95 % CI)	PPV (95 % CI)	NPV (95 % CI)	LR +ve (95 % CI)	LR −ve (95 % CI)	AUC (95 % CI)
Pain below knee	1	0.73 (0.66–0.78)	0.61 (0.55–0.66)	0.52 (0.46–0.58)	0.79 (0.74–0.84)	1.85 (1.58–2.18)	0.45 (0.35–0.58)	0.67 (0.62–0.72)
	2	0.68 (0.62–0.73)	0.68 (0.62–0.74)	0.72 (0.66–0.77)	0.65 (0.58–0.70)	2.13 (1.74–2.62)	0.47 (0.39–0.57)	0.68 (0.63–0.73)
Frequency of pain								
Nearly all the time/constant^a^	1	0.67 (0.60–0.74)	0.50 (0.44–0.55)	0.44 (0.39–0.50)	0.72 (0.66–0.78)	1.34 (1.16–1.55)	0.66 (0.52–0.83)	0.59 (0.53–0.64)
Constant^b^		0.24 (0.19–0.31)	0.79 (0.74–0.83)	0.41 (0.32–0.50)	0.64 (0.59–0.68)	1.16 (0.83–1.61)	0.96 (0.87–1.06)	0.52 (0.46–0.57)
Nearly all the time/constant^a^	2	0.65 (0.59–0.70)	0.53 (0.47–0.59)	0.62 (0.56–0.67)	0.56 (0.49–0.62)	1.37 (1.17–1.62)	0.67 (0.55–0.82)	0.59 (0.54–0.64)
Constant^b^		0.23 (0.19–0.29)	0.79 (0.73–0.84)	0.57 (0.47–0.65)	0.46 (0.42–0.51)	1.10 (0.79–1.53)	0.97 (0.89–1.07)	0.51 (0.46–0.56)
Severity of pain (NRS)^c^								
4–6^d^	1	0.87 (0.81–0.91)	0.23 (0.19–0.28)	0.40 (0.36–0.45)	0.74 (0.65–0.82)	1.12 (1.04–1.22)	0.58 (0.38–0.88)	0.55 (0.50–0.60)
7–10^e^		0.48 (0.41–0.55)	0.73 (0.68–0.78)	0.51 (0.44–0.59)	0.70 (0.65–0.75)	1.77 (1.40–2.24)	0.71 (0.61–0.83)	0.60 (0.55–0.66)
4–6^d^	2	0.87 (0.82–0.90)	0.26 (0.21–0.32)	0.58 (0.54–0.63)	0.62 (0.52–0.71)	1.17 (1.07–1.28)	0.52 (0.36–0.75)	0.56 (0.51–0.61)
7–10^e^		0.43 (0.37–0.49)	0.75 (0.69–0.80)	0.67 (0.60–0.74)	0.52 (0.47–0.58)	1.73 (1.33–2.25)	0.76 (0.67–0.86)	0.59 (0.54–0.64)
Which pain worst								
Both as bad^f^	1	0.69 (0.62–0.75)	0.52 (0.46–0.57)	0.46 (0.40–0.52)	0.74 (0.68–0.79)	1.44 (1.24–1.67)	0.59 (0.47–0.75)	0.61 (0.56–0.66)
Leg pain^g^		0.40 (0.34–0.47)	0.78 (0.73–0.82)	0.52 (0.44–0.60)	0.69 (0.64–0.73)	1.83 (1.39–2.39)	0.77 (0.67–0.87)	0.59 (0.54–0.64)
Both as bad^f^	2	0.70 (0.64–0.75)	0.60 (0.54–0.66)	0.67 (0.62–0.72)	0.62 (0.56–0.68)	1.73 (1.45–2.07)	0.51 (0.42–0.63)	0.65 (0.60–0.70)
Leg pain^g^		0.39 (0.33–0.45)	0.83 (0.78–0.88)	0.73 (0.66–0.80)	0.54 (0.48–0.59)	2.32 (1.68–3.20)	0.73 (0.66–0.82)	0.61 (0.56–0.66)
Coughing/sneezing	1	0.34 (0.28–0.41)	0.79 (0.74–0.83)	0.49 (0.41–0.57)	0.67 (0.62–0.72)	1.64 (1.22–2.19)	0.83 (0.74–0.94)	0.57 (0.51–0.62)
	2	0.30 (0.25–0.36)	0.80 (0.74–0.84)	0.63 (0.55–0.71)	0.49 (0.44–0.54)	1.48 (1.08–2.01)	0.88 (0.79–0.97)	0.55 (0.50–0.60)
Tingling	1	0.35 (0.29–0.42)	0.80 (0.75–0.84)	0.50 (0.42–0.59)	0.68 (0.63–0.72)	1.73 (1.29–2.32)	0.82 (0.73–0.92)	0.57 (0.52–0.63)
	2	0.31 (0.26–0.37)	0.80 (0.75–0.85)	0.65 (0.56–0.73)	0.50 (0.45–0.55)	1.57 (1.15–2.15)	0.86 (0.78–0.95)	0.56 (0.51–0.61)
Shooting pain	1	0.33 (0.27–0.40)	0.72 (0.67–0.76)	0.41 (0.33–0.48)	0.65 (0.59–0.69)	1.16 (0.89–1.52)	0.94 (0.83–1.06)	0.52 (0.47–0.58)
	2	0.34 (0.29–0.40)	0.75 (0.69–0.80)	0.62 (0.54–0.69)	0.49 (0.44–0.55)	1.40 (1.06–1.84)	0.87 (0.78–0.97)	0.55 (0.50–0.60)
Burning pain	1	0.27 (0.21–0.33)	0.84 (0.79–0.87)	0.49 (0.39–0.58)	0.66 (0.61–0.70)	1.61 (1.14–2.26)	0.88 (0.80–0.97)	0.55 (0.50–0.60)
	2	0.25 (0.20–0.30)	0.86 (0.81–0.90)	0.67 (0.57–0.75)	0.49 (0.44–0.54)	1.73 (1.19–2.51)	0.88 (0.81–0.96)	0.55 (0.50–0.60)
Sharp pain	1	0.38 (0.31–0.45)	0.74 (0.69–0.79)	0.46 (0.38–0.54)	0.67 (0.62–0.72)	1.46 (1.12–1.89)	0.84 (0.74–0.96)	0.56 (0.51–0.61)
	2	0.37 (0.31–0.42)	0.77 (0.72–0.82)	0.66 (0.58–0.73)	0.51 (0.46–0.56)	1.62 (1.22–2.15)	0.82 (0.73–0.92)	0.57 (0.52–0.62)
Toothache	1	0.47 (0.40–0.54)	0.53 (0.48–0.59)	0.37 (0.31–0.43)	0.63 (0.57–0.68)	0.99 (0.82–1.20)	1.01 (0.85–1.19)	0.50 (0.45–0.55)
	2	0.48 (0.42–0.53)	0.54 (0.48–0.60)	0.55 (0.49–0.61)	0.47 (0.41–0.53)	1.03 (0.86–1.24)	0.97 (0.83 –1.14)	0.51 (0.46–0.56)
Numbness, pins and needles	1	0.64 (0.56–0.70)	0.60 (0.55–0.65)	0.48 (0.42–0.55)	0.74 (0.68–0.79)	1.60 (1.34–1.90)	0.61 (0.49–0.75)	0.62 (0.57–0.67)
	2	0.59 (0.54–0.65)	0.64 (0.58–0.70)	0.66 (0.60–0.72)	0.57 (0.51–0.63)	1.66 (1.37–2.03)	0.63 (0.53–0.75)	0.62 (0.57–0.67)

Diagnostic classification: 1, non-neural and inconclusive versus neural (confirmatory diagnostic); 2, non-neural versus neural and inconclusive (indicative possible diagnostic)

^a^Reference = ‘Sometimes’

^b^Reference = ‘Sometimes’ or ‘Nearly all the time’)

^c^Severity of pain on a discrete 0–10 scale yielded an AUC of 0.624 (95 % CI 0.574–0.675)

^d^‘4–6’ or ‘7–10’ (reference = ‘0–3')

^e^‘7–10’ (reference = ‘0–3’ or ‘4–6')

^f^‘Both as bad’ *or* ‘Leg pain’ (reference = ‘Back pain’)

^g^‘Leg pain’ (reference = ‘Back pain’ or ‘Both as bad’)

Associations, both univariable and multivariable, between the self-report items and clinical diagnosis of nerve root involvement are shown in Table 5. In univariable testing, all items except ‘toothache’ (and ‘shooting pain’ in the confirmatory diagnostic classification) were significantly associated with the clinical diagnosis. However, only three variables were significantly independent items (at the level of *P* < 0.05) in the full multivariable analysis—‘pain below the knee’, ‘which pain worst’ (leg pain only versus not leg pain only) and ‘numbness, pins and needles’ (the latter being significant in respect of testing against indicative diagnostic classification). The item ‘coughing/sneezing’ was associated with the confirmatory diagnostic classification at the level of 0.05 < *P* < 0.1, but was excluded from the multivariable forward-selection model as it was not strongly associated with both diagnostic classifications. Diagnostic statistics for four multivariable models are shown in Table 6. Included is the full-entry model (which included all items) and three manual forward-selection models; the first being based on ‘pain below the knee’ only; the second, additionally including ‘which pain worst’ (leg pain only versus not leg pain only), and the third including all three aforementioned plus ‘numbness, pins & needles’. For the full model, the selected categorisation cut-off for ‘frequency of pain’ was ‘nearly all the time’; ‘severity of pain’ was ‘7–10’, and ‘which pain worst’ was ‘leg pain’ [on the basis that these gave highest odds ratios in the tests of association (Table 5)]. The full-model yields an AUC of 0.74 against the confirmatory classification and 0.76 against the indicative classification, and explains 23 and 27 % of the variance respectively. These are only marginally better fits than the forward-selection models that include the three most prognostic items (as shown in Model 2(iii), Table 6). The models were more specific than sensitive in relation to the confirmatory diagnostic reference, but were similarly sensitive and specific in relation to the indicative reference. Differences could also be seen in relation to predictive values, where the models were more likely to yield higher NPVs than PPVs with respect to the confirmatory diagnostic classification, whereas PPVs and NPVs were similar with respect to the indicative reference. Revised returns for these diagnostic statistics may be achieved by adjusting the classification cut-off from 0.5. Examples are illustrated in the legend of Table 6, and demonstrate that the sensitivity is increased and specificity decreased when the cut-off is lowered (e.g. to 0.3), whereas the opposite applies when the cut-off is raised above 0.5 (e.g. as shown by the figures for a 0.7 cut-off).

**Table 5:** Odds ratios (95 % CI) for associations between single items (univariable models) and multi-items (multivariable model) with clinic screening ‘gold-standard’

Question	Diagnostic classification 1 (confirmatory)				Diagnostic classification 2 (indicative possible)			
	Univariable		Multivariable		Univariable		Multivariable	
	OR (95 % CI)	*P* value	OR (95 % CI)	*P* value	OR (95 % CI)	*P* value	OR (95 % CI)	*P* value
Pain below knee	4.10 (2.78– 6.05)	<0.001	2.61 (1.68–4.06)	<0.001	4.56 (3.14–6.62)	<0.001	2.82 (1.85–4.29)	<0.001
Frequency of pain								
Sometimes^a^	–	–	–	–	–	–	–	–
Nearly all the time	2.23 (1.47–3.39)	<0.001	1.35 (0.83–2.18)	0.228	2.39 (1.58–3.59)	<0.001	1.34 (0.82–2.17)	0.244
Constant	1.76 (1.09–2.84)	0.020	1.35 (0.79–2.31)	0.271	1.64 (1.04–2.59)	0.034	1.18 (0.70–2.01)	0.535
Severity of pain (NRS)								
0–3^b^	–	–	–	–	–	–	–	–
4–6	1.33 (0.78–2.27)	0.293	0.99 (0.55–1.78)	0.973	1.72 (1.06–2.80)	0.028	1.26 (0.73–2.16)	0.406
7–10	3.05 (1.77–5.25)	<0.001	1.62 (0.85–3.11)	0.143	3.36 (2.00–5.63)	<0.001	1.49 (0.79–2.80)	0.221
Which pain worst								
Back pain^c^	–	–	–	–	–	–	–	–
Both as bad	1.89 (1.20–2.97)	0.006	1.04 (0.62–1.75)	0.886	2.57 (1.66–3.98)	<0.001	1.50 (0.91–2.46)	0.111
Leg pain	3.09 (1.99–4.80)	<0.001	1.99 (1.20–3.31)	0.008	4.54 (2.88–7.16)	<0.001	2.88 (1.72–4.82)	<0.001
Coughing/sneezing	1.96 (1.31–2.94)	0.001	1.51 (0.95–2.42)	0.084	1.68 (1.12–2.53)	0.012	1.23 (0.76–1.99)	0.410
Tingling	2.12 (1.42–3.18)	<0.001	1.44 (0.88–2.34)	0.147	1.83 (1.21–2.76)	0.004	1.21 (0.72–2.03)	0.476
Shooting pain	1.24 (0.84–1.83)	0.279	0.89 (0.56–1.41)	0.605	1.60 (1.09–2.36)	0.017	1.32 (0.83–2.10)	0.246
Burning pain	1.83 (1.18–2.83)	0.007	1.27 (0.77–2.10)	0.344	1.97 (1.25–3.10)	0.003	1.47 (0.86–2.49)	0.156
Sharp pain	1.73 (1.18–2.55)	0.005	1.11 (0.69–1.79)	0.667	1.98 (1.34–2.93)	0.001	1.29 (0.79–2.10)	0.314
Toothache	0.99 (0.69–1.41)	0.942	1.06 (0.68–1.64)	0.801	1.06 (0.75–1.51)	0.734	1.35 (0.86–2.11)	0.191
Numbness, pins and needles	2.64 (1.82–3.82)	<0.001	1.50 (0.95–2.35)	0.079	2.63 (1.84–3.77)	<0.001	1.68 (1.07–2.63)	0.024

^a^Reference category = ‘Sometimes’

^b^Reference category = ‘0–3’

^c^Reference category = ‘Back pain (only)’

**Table 6:** Discriminate statistics for combined self-report multi-item decision models against clinic screening ‘reference-standard’

Model reference	Sensitivity (95 % CI)	Specificity (95 % CI)	PPV (95 % CI)	NPV (95 % CI)	LR +ve (95 % CI)	LR −ve (95 % CI)	AUC (95 % CI)	*R* ^2b^
Diagnostic classification 1: confirmatory								
Model 1 (full entry)	0.52 (0.45–0.59)	0.82 (0.77–0.86)	0.63 (0.56–0.71)	0.74 (0.69–0.78)	2.88 (2.19–3.79)	0.59 (0.50–0.69)	0.74 (0.70–0.79)	0.23
Model 2 [forward-selection (step)]								
2(i) Pain below the knee (only)	0.73 (0.66–0.78)	0.61 (0.55–0.66)	0.52 (0.46–0.58)	0.79 (0.74–0.84)	1.85 (1.58–2.18)	0.45 (0.35–0.58)	0.67 (0.62–0.72)	0.14
2(ii) + leg pain worst	0.33 (0.27–0.40)	0.90 (0.86–0.93)	0.66 (0.56–0.75)	0.69 (0.65–0.74)	3.31 (2.25–4.87)	0.74 (0.67–0.83)	0.70 (0.65–0.75)	0.17
2(iii) + numbness, pain and needles^a^	0.59 (0.52–0.66)	0.75 (0.70–0.80)	0.59 (0.52–0.65)	0.76 (0.71–0.80)	2.39 (1.91–2.99)	0.54 (0.45–0.65)	0.72 (0.67–0.77)	0.19
Diagnostic classification 2: Indicative possible								
Model 1 (full entry)	0.73 (0.68–0.78)	0.65 (0.59–0.71)	0.72 (0.66–0.77)	0.67 (0.60–0.73)	2.09 (1.73–2.54)	0.41 (0.33–0.51)	0.76 (0.72–0.80)	0.27
Model 2 [forward-selection (step)]								
2(i) Pain below the knee (only)	0.68 (0.62–0.73)	0.68 (0.62–0.74)	0.72 (0.66–0.77)	0.65 (0.58–0.70)	2.13 (1.74–2.62)	0.47 (0.39–0.57)	0.68 (0.63–0.73)	0.17
2(ii) + leg pain worst	0.78 (0.73–0.83)	0.58 (0.52–0.64)	0.69 (0.64–0.74)	0.69 (0.62–0.75)	1.87 (1.59–2.20)	0.38 (0.29–0.48)	0.73 (0.68–0.77)	0.22
2(iii) + numbness, pain and needles^a^	0.73 (0.67–0.78)	0.66 (0.60–0.72)	0.72 (0.66–0.77)	0.67 (0.61–0.73)	2.14 (1.76–2.59)	0.41 (0.33–0.51)	0.74 (0.70–0.79)	0.24

+ indicates the variable has been added manually to the forward-selection model

^a^Modification of the classification cut-off (from the default of 0.5) for neural diagnosis leads to changes in the diagnostic statistics of sensitivity, specificity, PPV, NPV, LR+ and LR− respectively (without changing the model fit), as follows:-

Cut-off // Confirmatory classification // Indicative classification

0.3 // 0.75; 0.57; 0.51; 0.79; 1.74; 0.44 // 0.86; 0.40; 0.63; 0.70; 1.42; 0.36

0.7 // 0.00; 1.00; 0.00; 0.62; 0.00; 1.00 // 0.54; 0.82; 0.78; 0.60; 2.99; 0.56

^b^Nagelkerke *R* ^2^ (percent variance explained)

## Discussion

In this study we explored whether self-report of symptoms is an accurate way for defining sciatica cases. The results suggest that self-reported items alone are not sufficiently accurate in selecting subjects with sciatica in epidemiological studies.

Pain below knee was the best single item for diagnostic accuracy with an AUC of 0.67 which however is less than ‘acceptable discrimination’. In this cohort, the commonly used (or suggested) proxies of ‘pain radiating to the legs’ [9], or ‘below the knee’ [11, 13] overestimate the prevalence by 170 and 39 % respectively. In contrast, ‘pain radiating to the leg(s) that worsens with coughing and sneezing’ [15] underestimates the prevalence by 31 %. Sensitivity and specificity ranges were wide ranging across the individual self-report items, but the discriminative ability of the individual items were low as reflected by the positive and negative likelihood ratios falling mostly under 2 and above 0.5, respectively.

The individual single-items were not independent in their predictive capacity (Table 4). Though, a cluster of three self-reported items, including distribution of pain below the knee, leg pain that is worse and feeling of numbness or pins and needles in the leg, did improve discrimination to an ‘acceptable’ level with an AUC of 0.72 in respect of the confirmatory diagnostic reference and 0.74 in respect of the indicative reference. However, the likelihood ratios from the model [model 2(iii)] were indicative of a ‘small’ amount of discrimination. Approximately half of all clinically confirmed sciatica cases were misclassified as non-cases. As indicated by the higher NPV of 0.76 compared to the PPV of 0.59, a negative test result [according to the three-item model 2(iii)] was more likely to predict absence of nerve root involvement than a positive test result was to truly predict presence of nerve root involvement—in relation to clear cases of nerve root involvement (as based on the confirmatory classification). Sensitivity and specificity was similar at about 0.7 when the self-report models were tested against the less strict indicative/possible diagnostic criteria; the PPV and NPV values also providing similar probabilities of about 0.7 for correct predictions. However, irrespective of which diagnostic classification is of interest, the findings indicate that the probability of false test results would be high overall.

Vroomen et al. [24] previously reported on the contribution of history and examination items to the diagnosis of lumbar radiculopathy due to a disc prolapse and suggested that examination items contribute little after the establishment of the history items. History items are mainly self-reported but in the context of the clinical examination clarification can be obtained which most likely improves accuracy of reporting. In addition, this study’s [24] population was a highly selected cohort of patients, a factor that is likely to contribute to high diagnostic accuracy as patients tend to be at the worse end of the spectrum.

Our findings suggest that when the objective of a study is to capture the presence of back pain that spreads to the leg(s), as perhaps an index of severity, then proxies such as ‘pain down the leg’ or ‘pain below the knee’ may be acceptable but for specific presentations such as sciatica such proxies do not seem sufficiently accurate. Even with a cluster of positive symptoms [Models 1 and 2(iv)] the discrimination although acceptable may still be considered problematic as indicated by the low sensitivity estimates.

A number of points pertaining to strengths and limitations of this study merit some further discussion. Firstly, the self-reported items selected as potential indicators of sciatica. We believe that these are in accordance with current literature as potentially contributing to the clinical diagnosis or impression of the presence or absence of sciatica.

Second point is the acceptance of the clinical judgement by the assessing physiotherapists as the ‘reference standard’. The absence of a ‘gold reference standard’ in sciatica is well documented in the relevant literature [7, 25] and clinical diagnosis does serve as the ‘reference standard’ in a number of studies [25]. Taking into account that patients with positive imaging findings of nerve root involvement may be asymptomatic and vice versa and that in primary care, at least initially, diagnosis is based on clinical assessment alone, it is reasonable to use clinical diagnosis as the ‘reference standard’ in primary care as opposed to imaging tests. However, misdiagnosis of cases is possible.

A third point pertains to the assessors in this study. A large number of assessors participated and although this may introduce variability it also contributes to the generalisability of results. Nevertheless, all assessors underwent training and all clinical assessment information was collected in a standardised manner. All assessors were physiotherapists and one may argue that medically trained clinicians (general practitioners for example) may be better in diagnosing the presence or absence of nerve root involvement in patients with low back and leg pain, and therefore diagnostic accuracy of items could vary if diagnosis varied. However, to our knowledge, there is no evidence in the published literature to suggest that there is a difference between different health care professionals although there may be differences depending on level of clinical experience.

Fourth point is the population studied and the method. This was a truly primary care population presenting with variable severity and duration of symptoms. There was no selection bias towards the worse cases. Patients were asked on the day to answer the set of questions literature suggests be included in the assessment of symptoms of LBP with leg pain to assess probability of nerve root involvement. The patients were asked to think about their symptoms and symptom behaviour within the last week. There is a possibility that with asking about symptoms within the last week some patients, although having had these symptoms to a greater or a lesser degree, may have substantially recovered on the day of assessment and therefore findings of clinical history and examination were negative leading to decreased discrimination values. We do not know if results would be different in a secondary care population for example in which case one expects more severe symptoms which may be easier to recognise. This though contributes to the problem of selection bias.

## Conclusion

Low back pain with leg pain is a common presentation, and in a number of cases the presence of leg pain is due to a spinal nerve root involvement causing radiculopathy (sciatica). Self-reported sciatica or indicators suggestive of sciatica have been used in studies for capturing the prevalence of the condition or for exploring risk factors for the onset or persistence. This is the first study to investigate the diagnostic accuracy of commonly used patient self-report items for sciatica in a primary care setting and in an unselected population presenting with LBP and leg pain. The results suggest that self-report is not an accurate method for identifying individuals with the condition and it may overestimate or underestimate its prevalence. Certain self-report indicators particularly pain radiating below, leg pain worse than back pain and numbness, pins and needles in the leg can be useful at a very crude level. However, when accuracy in case definition is of importance, clinical examination is the recommended method.

## Footnotes

1 Classification 1: no nerve root pain and inconclusive cases versus nerve root pain (confirmatory reference).

2 Classification 2: no nerve root pain versus nerve root pain and inconclusive cases (indicative reference).
